# Efficacy of Traditional Herbal Medicine Treatment Based on Pattern Identification for Idiopathic Parkinson's Disease: A Protocol for Systematic Review and Meta-Analysis

**DOI:** 10.1155/2022/4777849

**Published:** 2022-04-30

**Authors:** Purumea Jun, HuiYan Zhao, Ojin Kwon, Jung-Hee Jang

**Affiliations:** ^1^KM Science Research Division, Korea Institute of Oriental Medicine, 1672 Yuseongdae-ro, Yuseong-gu, Daejeon 34054, Republic of Korea; ^2^University of Science & Technology, Campus of Korea Institute of Oriental Medicine, Korean Convergence Medicine Major, 217 Gajeong-ro Yuseong-gu, Daejeon, Republic of Korea

## Abstract

Parkinson's disease (PD), the second most common progressive neurodegenerative disease, is characterized by various clinical symptoms and reduced quality of life. The standard dopaminergic therapy for PD has limitations such as drug wear-off, drug-related side effects, and drug-resistant PD symptoms. Traditional oriental medicine, which is a personalized approach based on pattern identification (PI), has been reported to relieve symptoms, halt disease progression, and improve the quality of life in patients with PD. This comprehensive systematic review will be conducted to gather clinical studies related to complementary traditional herbal therapies based on PI for idiopathic PD and assess its effectiveness. Clinical studies, including randomized controlled trials in English, Korean, and Chinese databases related to the efficacy of herbal medicine based on PI for PD will be searched in computer retrieval. In addition, the subdivided PI for each clinical manifestation of PD will be investigated. Two researchers will independently screen and select studies, extract data, and assess bias risk. The risk of bias will be evaluated using the Cochrane risk-of-bias assessment tool. After screening the studies, a meta-analysis will be performed. The primary outcome will be the unified Parkinson's disease rating scale to measure clinical symptom reduction. Secondary outcomes will consist of other validated scales to evaluate the improvement of PD, including improvement of clinical symptoms and quality of life. The quality of evidence will be evaluated through the Grading of Recommendations, Assessment, Development, and Evaluation pro. Complementary traditional medicine is a personalized medicine that classifies individual states based on PI. We expect that the results of this review will provide evidence for the efficacy of traditional herbal medicine based on PI for the treatment of PD. This protocol has been registered in the International Platform of Registered Systematic Review and Meta-Analysis Protocols (INPLASY) 2021 (registration number INPLASY2021100020).

## 1. Introduction

Parkinson's disease (PD) is the second most common neurodegenerative disorder, and its global prevalence increases sharply with age. The neuropathology of PD is characterized by striatal dopamine deficiency due to neuronal loss in the substantia nigra [1, 2]. The clinical symptoms of patients with PD include motor manifestations, such as tremor, rigidity, bradykinesia, postural instability, and gait disorders, and nonmotor manifestations involving a multitude of functions, such as disorders of sleep-wake cycle regulation, cognitive impairment, disorders of mood and affect, autonomic dysfunction, as well as sensory symptoms and pain [1]. Patients with PD suffer from complex symptoms and experience a reduced quality of life [1, 2]. Although dopaminergic medications are the standard therapy for drastic improvement of the motor symptoms and quality of life in patients with PD, once the “honeymoon” period of the drugs has waned, patients with PD become progressively more disabled due to the wearing-off of levodopa effects caused by long-term administration [3–5]. Moreover, dopa-resistant PD symptoms include motor signs such as posture, gait, and balance problems, and nonmotor signs such as autonomic dysfunction, mood and cognitive impairment, sleep problems, pain, and drug-related adverse effects such as psychosis, motor fluctuations, and dyskinesia [5]. In addition, therapies to effectively slow down or halt PD progression are not available [6]. Therefore, complementary and alternative treatments are required to overcome the limitations of dopaminergic medications.

Furthermore, the pathophysiology of PD involves multiple neurotransmitter deficiencies according to multisystem neurodegeneration, which contributes to the complexity of broad-spectrum clinical symptoms [7, 8]. The clinical symptoms of PD vary widely, and individual medicine is needed for a personalized approach for each patient from a holistic perspective [8, 9]. For personalized medicines, the subtype classification of PD is gaining attention in the West [10]. In a previous study, the subtype classifications of PD according to the age of onset, clinical phenotypes, and disease severity or neuropathological alterations have been reported [8]. In addition, recent research on classification using clinical phenotypes in PD has reported subtyping after comprehensively considering dominance, severity, and prognosis of motor and nonmotor symptoms and biomarker development [9, 11, 12].

Traditional oriental medicine, an ancient system of personalized medicine, classifies individual states based on the analysis of a symptom complex or cluster [13–15]. It is a holistic system of medicine that involves treatment based on pattern identification (PI), a unique theory of diagnosis [16]. After PI, patients are prescribed a customized treatment according to their specific condition. Traditional medicine theories have been used to identify patterns to treat PD, relieve symptoms, halt disease progression, and improve quality of life [17]. Traditional medicine treatment of PD, according to PI, has been reported to improve the clinical symptoms and reduce complications when compared to fixed recipe treatment of traditional medicine [18]. The effectiveness of the therapy is believed to be largely dependent on whether the treatment is in accordance with the rightly diagnosed pattern [19].

The first aim of this study is to conduct a systematic review to assess the efficacy of personalized traditional herbal medicine based on PI in PD. We will explore the possibility of overcoming the limitations of standard dopaminergic medications. The second aim is to conduct a systematic review to investigate the most frequent pattern and symptoms while treating PD with traditional herbal medicine through electronic searches of journal articles from Korean, Chinese, and English databases. Finally, by investigating the subdivided PI for each clinical symptom of PD, we will suggest a more detailed and customized treatment for PD.

## 2. Materials and Methods

### 2.1. Study Registration

This systematic review protocol adheres to the Preferred Reporting Items for Systematic Reviews and Meta-Analyses Protocols (PRISMA-P) statement guidelines [20]. This protocol has been registered in the International Platform of Registered Systematic Review and Meta-Analysis Protocols (INPLASY) 2021 (registration number INPLASY2021100020; https://inplasy.com/inplasy-2021-10-0020/). The PRISMA-P checklist is provided in Table S1.

### 2.2. Eligibility Criteria

#### 2.2.1. Criteria for Inclusion and Exclusion

The systematic review and meta-analysis will include all randomized controlled trials published until August 2021 in English, Korean, or Chinese. The searched studies have classified the PI and evaluated the efficacy of traditional medicine for PD, and any type of control intervention will be included. Observational studies, single case reports, literature reviews, republished research papers, recited literature, and other studies that fail to meet the inclusion criteria will be excluded.

### 2.3. Participants

Participants in this study will be those diagnosed with idiopathic PD by a physician based on the UK Parkinson's Disease Society Brain Bank Criteria, as well as participants classified based on PI. In addition, patients will be included with no restrictions on other conditions, such as age, sex, country of origin, or severity of symptoms.

### 2.4. Types of Interventions

The interventions will include traditional herbal medications prescribed after PI, regardless of the formulae, form of administration, dosage, frequency, or duration of treatment, in combination with conventional medications, physical therapy, or other therapies, or alone. Only studies with the administration of traditional herbal medicine according to the pattern diagnosed will be included.

### 2.5. Types of Comparisons

Trials with any type of comparator will be included.

### 2.6. Types of Outcomes

The primary outcome measure will be the total score of the unified Parkinson's disease rating scale (UPDRS) assessment. Secondary outcome measures will include indicators of improvement in clinical symptoms of PD, except for primary outcome (e.g., the partial scores of UPDRS part assessment, PD questionnaire-39, nonmotor symptoms scale for PD, and PD sleep scale).

### 2.7. Search Strategies for Data Sources

Databases and search terms will be determined through discussions among all authors before the literature search is executed. The following electronic databases will be searched from inception to August 2021, regardless of the publication status: three core databases (MEDLINE, Embase, and Cochrane Library), three Korean databases (Oriental Medicine Advanced Searching Integrated System, Korean Studies Information Service System, and Korea Citation Index), and four Chinese databases (China National Knowledge Infrastructure, China Science and Technology Journal Database, WanFang database, and China Biology Medicine disc). The search strategy for PubMed is shown in Table 1 and that for other databases is shown in Table S2.

### 2.8. Data Collection and Screening

#### 2.8.1. Study Selection

Three authors (PJ, HYZ, and JHJ) will independently search the databases and assess potentially relevant articles against the inclusion criteria. The title, author, and journal name/year/issue of the retrieved papers will be imported into EndNoteTM 20.1 to determine the relevant studies. When there are several similar research papers, only one paper will be included. The title and abstract of each paper will be read to exclude nonrelevant papers based on the inclusion and exclusion criteria. Any disagreement regarding the eligibility of the study will be resolved by consensus. In line with the PRISMA guidelines, the flow diagram of the study selection process is shown in Figure S1.

#### 2.8.2. Data Extraction

The three authors will independently extract the data in an Excel spreadsheet. For each study, the following variables will be extracted: author information, year of publication, study design, treatment regimen and control intervention, sample size, inclusion and exclusion criteria, participants' characteristics (age and sex, among others), information regarding the PI (symptoms and signs of each PI and number of subjects belonging to each pattern, among others), and primary and secondary outcome measurements. All Korean or Chinese to English translations will be deduced primarily from the World Health Organization's international standard terminologies. Furthermore, any discrepancy in the cross-checking process will be resolved by discussion.

### 2.9. Statistical Analysis

#### 2.9.1. Assessment of Risk of Bias

Using a previously standardized and reliable bias assessment tool, the external and internal validity of the studies will be assessed by two investigators. Based on the overall bias assessment score, articles will be divided into low, moderate, or high risk of bias. Any uncertainty or disagreement between the investigators will be resolved by discussion between two authors. If no consensus can be reached, a third investigator will review the study to break the impasse.

### 2.10. Measures of Treatment Effect

The Review Manager (version 5.4) software of the Cochrane Collaboration will be used for data analysis. Data for binary outcomes will be summarized using odds ratios with 95% confidence intervals (CIs), and data for continuous outcomes will be summarized using mean difference (MD) or standardized mean difference (SMD) with 95% CI.

### 2.11. Dealing with Missing Data

If there are missing data, the authors will try to obtain the necessary information by contacting the first or corresponding authors of the included trials by phone, e-mail, or fax.

### 2.12. Assessment of Heterogeneity

Heterogeneity among trials will be detected using both the *χ*2 and the I^2^ statistic. If the I^2^ value is greater than 50%, it will be considered indicative of substantial heterogeneity. If there is substantial heterogeneity, subgroup analysis stratified by different control groups will be performed to look for explanations for the heterogeneity.

### 2.13. Data Synthesis

For continuous variables (UPDRS and indicators of improvement in clinical symptoms of PD), we will use mean (including MD or SMD) and standard deviation (SD) and a 95% CI in the meta-analysis. Dichotomous results will be expressed as relative risk with 95% CI. As aforementioned, we will use I2 and *χ*2 teat to analyze statistic heterogeneity. If *p* > 0.1 and I2 < 50%, which means homogeneity, a fixed-effects model will be used in the meta-analysis; otherwise, if *p* > 0.1 and I2 > 50%, the random-effects model will be conducted and subgroup analysis will be performed to explore the possible reason for heterogeneity. A narrative synthesis will be provided if the meta-analysis cannot be performed for all or some of the expected data from the included studies.

If enough trials are identified, subgroups of different types of clinical symptoms in PD will be analyzed separately (e.g., tremor).

### 2.14. Sensitivity Analysis

If there are a high-quality methodology, sufficient sample size, and low heterogeneity, a sensitivity analysis will be carried out to analyze the robustness of the study.

### 2.15. Grading the Quality of Evidence

The quality of evidence for all outcomes will be working group methodology across the domains of risk of bias, consistency, directness, precision, and publication bias assessed by the Grading of Recommendations, Assessment, Development, and Evaluation pro [21]. The quality of evidence will be evaluated as high, moderate, low, or very low.

### 2.16. Assessment of Reporting Biases

If the number of final screening studies included in the meta-analysis exceeds 10, a funnel plot will be used to detect whether reporting bias exists.

### 2.17. Ethics and Dissemination

This study does not require ethical approval because it is based on a review of published literature and does not involve any private data. The final reports of this study will be published in a peer-reviewed journal and disseminated electronically and in print.

## 3. Discussion

PD is associated with clinical phenotyping variability involving the pathophysiology of multiple neurotransmitter deficiencies, resulting in multisystem neurodegeneration [7, 8]. Moreover, the multiple clinical symptoms of PD are associated with disease progression [1]. Therefore, PD treatment strategies should consider a number of factors, including not only the cross-sectional but also the longitudinal dimensions of disease progression. Therefore, treatments must be tailored to individual patients or disease progression in the same patient. Several studies on classification for subtyping PD based on clinical, pathological, genetic, and molecular features, as well as biomarkers to distinguish individual subtypes, should be developed [22]. However, it is currently difficult to present a subtype with obvious criteria and guide personalized treatment for individual patients with PD. Traditional oriental medicine classifies individual states based on the analysis of a symptom complex or cluster and provides personalized therapies [13–15]; however, a systematic review of the efficacy of traditional therapies based on PI in PD has not yet been reported.

## 4. Conclusions

To the best of our knowledge, this will be the first systematic review to assess the efficacy of traditional herbal medicine based on PI in PD, which is intended to provide complementary personalized treatment strategies to benefit clinical practitioners and patients with PD.

## Figures and Tables

**Table 1:** The search strategy for PubMed.

#1	“Parkinson's disease” [MeSH terms]
#2	“parkinson”$ [Title/Abstract]
#3	“parkinson's disease” [Title/Abstract]
#4	“parkinsons disease” [Title/Abstract]
#5	“parkinsonism disease” [Title/Abstract]
#6	“paralysis agitans” [Title/Abstract]
#7	(((((#1) OR (#2)) OR (#3)) OR (#4)) OR (#5)) OR (#6)
#8	“pattern” [Title/Abstract]
#9	“syndrome” [Title/Abstract]
#10	“pattern identification” [Title/Abstract]
#11	“syndrome identification” [Title/Abstract]
#12	“pattern differentiation” [Title/Abstract]
#13	“syndrome differentiation” [Title/Abstract]
#14	“zheng” [Title/Abstract]
#15	“pattern medicine” [Title/Abstract]
#16	“syndrome pattern” [Title/Abstract]
#17	“Traditional Chinese medicine pattern” [Title/Abstract]
#18	“Traditional Chinese medicine syndrome” [Title/Abstract]
#19	“deficiency of Qi Blood” [Title/Abstract]
#20	“Yin deficiency of liver kidney” [Title/Abstract]
#21	“Phlegm Heat Wind stirring” [Title/Abstract]
#22	“Blood stasis Wind stirring” [Title/Abstract]
#23	“Qi stagnation Blood stasis” [Title/Abstract]
#24	“deficiency of Yin Yang” [Title/Abstract]
#25	#8 OR #9 OR #10 OR #11 OR #12 OR #13 OR #14 OR #15 OR #16 OR #17 OR #18 OR #19 OR #20 OR #21 OR #22 OR #23 OR #24
#26	#25 AND #7
#27	“herbal medicine” [Title/Abstract]
#28	“Korea medicine” [Title/Abstract]
#29	“phytotherapy” [MeSH terms]
#30	“ethnobotany” [Title/Abstract]
#31	“ethnopharmacology” [Title/Abstract]
#32	“Traditional Chinese medicine” [Title/Abstract]
#33	“Chinese medicine” [Title/Abstract]
#34	“herbal formula” [Title/Abstract]
#35	“herbal preparation” [Title/Abstract]
#36	“decoction” [Title/Abstract]
#37	#27 OR #28 OR #29 OR #30 OR #31 OR #32 OR #33 OR #34 OR #35 OR #36
#38	#37 AND #26

## Data Availability

The data are available from the authors upon reasonable request.

## Supplementary Materials

Supplementary Materials
*Table S1*. PRISMA-P (Preferred Reporting Items for Systematic Review and Meta-Analysis Protocols) 2015 checklist: recommended items to address in a systematic review protocol. *Table S2*. Search strategy for databases other than PubMed. *Figure S1*. PRISMA flow diagram of the study selection process. .
