# Supplementary material for: Efficacy of Traditional Herbal Medicine Treatment Based on Pattern Identification for Idiopathic Parkinson's Disease: A Protocol for Systematic Review and Meta-Analysis
Source: Evid Based Complement Alternat Med. 2022 Apr 30;2022:4777849. doi: 10.1155/2022/4777849 (PMC9078772; doi:10.1155/2022/4777849)
Supplement: Supplementary Materials — Table S1. PRISMA-P (Preferred Reporting Items for Systematic Review and Meta-Analysis Protocols) 2015 checklist: recommended items to address in a systematic review protocol. Table S2. Search strategy for databases other than PubMed. Figure S1. PRISMA flow diagram of the study selection process. . [file 4777849.f1.zip › 4777849.f1/4._Table_S2_R2_2022_0401.docx]

**Table S2: Search strategy for databases other than PubMed**

1. EMBASE

| #1 | “parkinson disease”/exp |
| --- | --- |
| #2 | “parkinson*” :ab, ti |
| #3 | “paralysis agitans” :ab, ti |
| #4 | “parkinsonism” |
| #5 | “parkinson disease” :ab, ti |
| #6 | #1 OR #2 OR #3 OR #4 OR #5 |
| #7 | “pattern*” |
| #8 | “syndrome*” |
| #9 | “pattern identification” :ab, ti |
| #10 | “syndrome identification” :ab, ti |
| #11 | “pattern differentiation” :ab, ti |
| #12 | “syndrome differentiation” :ab, ti |
| #13 | “zheng” |
| #14 | “pattern medicine” :ab, ti |
| #15 | “syndrome pattern” :ab, ti |
| #16 | “traditional Chinese medicine pattern” :ab, ti |
| #17 | “traditional Chinese medicine syndrome” :ab, ti |
| #18 | “syndrome” :ab, ti |
| #19 | “pattern” :ab, ti |
| #20 | “zheng” :ab, ti |
| #21 | “traditional Chinese medicine” :ab, ti |
| #22 | “Chinese medicine” :ab, ti |
| #23 | “herbal formula” :ab, ti |
| #24 | “herbal medicine” :ab, ti |
| #25 | “herbal decoction” :ab, ti |
| #26 | “herbal preparation” :ab, ti |
| #27 | “decoction” :ab, ti |
| #28 | “Korea medicine” :ab, ti |
| #29 | “traditional medicine” :ab, ti |
| #30 | “Chinese herbal” :ab, ti |
| #31 | “Korea herbal” :ab, ti |
| #32 | “herb” :ab, ti |
| #33 | “plants” :ab, ti |
| #34 | “ethnobotany” :ab, ti |
| #35 | “ethnopharmacology” :ab, ti |
| #36 | “phytotherapy” :ab, ti |
| #37 | “integrative medicine” :ab, ti |
| #38 | “Yin deficiency of liver” :ab, ti AND “kidney” :ab, ti |
| #39 | “deficiency of Qi” :ab, ti AND “Blood: :ab, ti |
| #40 | “Phlegm heat” :ab, ti AND “Wind stirring” :ab, ti |
| #41 | “Blood stasis” :ab, ti AND “Wind stirring” :ab, ti |
| #42 | “Qi stagnation” :ab, ti AND “Blood stasis:ab, ti |
| #43 | “deficiency of Yin” :ab, ti AND “Yang” :ab, ti |
| #44 | #7 OR #8 OR #9 OR #10 OR #11 OR #12 OR #13 OR #14 OR #15 OR #16 OR #17 OR #38 OR #39 OR #40 OR #41 OR #42 OR #43 |
| #45 | #6 AND #44 |
| #46 | #21 OR #22 OR #23 OR #24 OR #25 OR #26 OR #27 OR #28 OR #29 OR #30 OR #31 OR #32 OR #33 OR #34 OR #35 OR #36 OR#37 |
| #47 | #45 AND #46 |

2. Cochrane library

| #1 | MeSH descriptor: [“parkinson disease”] explode all trees |
| --- | --- |
| #2 | “parkinson*” |
| #3 | “paralysis agitans” |
| #4 | “parkinson disease” |
| #5 | “parkinsonism” |
| #6 | #1 OR #2 OR #3 OR #4 OR #5 |
| #7 | “pattern*” |
| #8 | “syndrome*” |
| #9 | “pattern identification” |
| #10 | “syndrome identification” |
| #11 | “pattern differentiation” |
| #12 | “syndrome differentiation” |
| #13 | “zheng” |
| #14 | “pattern medicine” |
| #15 | “syndrome pattern” |
| #16 | “traditional Chinese medicine pattern” |
| #17 | “traditional Chinese medicine syndrome” |
| #18 | “Yin deficiency of liver and kidney” |
| #19 | “deficiency of Qi and Blood” |
| #20 | “Phlegm heat and Wind stirring” |
| #21 | “Blood stasis and Wind stirring” |
| #22 | “Qi stagnation and Blood stasis” |
| #23 | “deficiency of Yin and Yang” |
| #24 | #7 OR #8 OR #9 OR #10 OR #11 OR #12 OR #13 OR #14 OR #15 OR #16 OR #17 OR #18 OR #19 OR #20 OR #21 OR #22 OR #23 |
| #25 | #24 AND #6 |
| #26 | “herbal medicine” |
| #27 | “traditional Chinese medicine” |
| #28 | “herbal formula” |
| #29 | “herbal decoction” |
| #30 | “herbal preparation” |
| #31 | “decoction” |
| #32 | “phytotherapy” |
| #33 | “Korea medicine” |
| #34 | “ethnobotany” |
| #35 | “ethnopharmacology” |
| #36 | “herb*” |
| #37 | #26 OR #27 OR #28 OR #29 OR #30 OR #31 OR #32 OR #33 OR #34 OR #35 OR #36 |
| #38 | #37 AND #25 |

3. CNKI (the Chinese National Knowledge Infrastructure)

| #1 | 主题：“帕金森” |
| --- | --- |
| #2 | 主题：“颤证” |
| #3 | 主题：“震颤” |
| #4 | 主题：“parkinson” |
| #5 | 主题：“中医” |
| #6 | #1 OR #2 OR #3 OR #4 AND #5 |

4. VIP (Technology Journal Database)

| #1 | 题名或关键词 =”帕金森病” OR ”parkinson disease” OR”parkinsonian” OR”帕金森氏病” OR”帕金森综合症” OR”帕金森氏综合症” OR”帕金森症” OR ”帕金森氏综合征” OR”震颤麻痹” OR”帕金森综合征” OR”帕金森氏症” OR”帕金森” |
| --- | --- |
| #2 | 题名或关键词 = 中医 + 中医药 + 中药 |
| #3 | #1 OR #2 |

5. WanFang

| （题名或关键词:“帕金森” OR 题名或关键词:“颤证” OR 题名或关键词:“Parkinson” or 题名或关键词: “震颤”） AND (题名或关键词:“中医”) |
| --- |

6. CBMdisc (China Biology Medicine disc)

| "帕金森"[常用字段:智能] AND "中医"[常用字段:智能] |
| --- |

7.OASIS (The Oriental Medicine Advanced Searching Integrated System)

| “파킨슨” |
| --- |

8.KISS (The Korean Studies Information Service System)

| “파킨슨” |
| --- |

9.KCI (Korea Citation Index)

| “파킨슨” |
| --- |
