# Supplementary material for: Efficacy of Traditional Herbal Medicine Treatment Based on Pattern Identification for Idiopathic Parkinson's Disease: A Protocol for Systematic Review and Meta-Analysis
Source: Evid Based Complement Alternat Med. 2022 Apr 30;2022:4777849. doi: 10.1155/2022/4777849 (PMC9078772; doi:10.1155/2022/4777849)
Supplement: Supplementary Materials — Table S1. PRISMA-P (Preferred Reporting Items for Systematic Review and Meta-Analysis Protocols) 2015 checklist: recommended items to address in a systematic review protocol. Table S2. Search strategy for databases other than PubMed. Figure S1. PRISMA flow diagram of the study selection process. . [file 4777849.f1.zip › 4777849.f1/5._Figure S1 (1).docx]

**Figure S1:** PRISMA flow diagram of the study selection process

**Identification of studies via databases and registers**

Records removed *before screening*:

Duplicate records removed

(n = )

Records marked as ineligible by automation tools (n = )

Records removed for other reasons (n = )

Records identified from*:

Databases (n = )

Registers (n = )

**Identification**

Records screened

(n = )

Records excluded**

(n = )

Reports sought for retrieval

(n = )

Reports not retrieved

(n = )

**Screening**

Reports assessed for eligibility

(n = )

Reports excluded:

Reason 1 (n = )

Reason 2 (n = )

Reason 3 (n = )

etc.

Studies included in review

(n = )

Reports of included studies

(n = )

**Included**

*Consider, if feasible to do so, reporting the number of records identified from each database or register searched (rather than the total number across all databases/registers).

**If automation tools were used, indicate how many records were excluded by a human and how many were excluded by automation tools.

*From:*  Page MJ, McKenzie JE, Bossuyt PM, Boutron I, Hoffmann TC, Mulrow CD, et al. The PRISMA 2020 statement: an updated guideline for reporting systematic reviews. BMJ 2021;372:n71. doi: 10.1136/bmj.n71

For more information, visit: <http://www.prisma-statement.org/>
