# Supplementary material for: Efficacy of Traditional Herbal Medicine Treatment Based on Pattern Identification for Idiopathic Parkinson's Disease: A Protocol for Systematic Review and Meta-Analysis
Source: Evid Based Complement Alternat Med. 2022 Apr 30;2022:4777849. doi: 10.1155/2022/4777849 (PMC9078772; doi:10.1155/2022/4777849)
Supplement: Supplementary Materials — Table S1. PRISMA-P (Preferred Reporting Items for Systematic Review and Meta-Analysis Protocols) 2015 checklist: recommended items to address in a systematic review protocol. Table S2. Search strategy for databases other than PubMed. Figure S1. PRISMA flow diagram of the study selection process. . [file 4777849.f1.zip › 4777849.f1/Certificate of English editing.pdf]

editage

Editing Certificate

This document certifies that the manuscript listed below has been edited to ensure language and grammar accuracy and is error free in these aspects. The logical presentation of ideas and the structure of the paper were also checked during the editing process. The edit was performed by professional editors at Editage, a division of Cactus Communications. The author's core research ideas were not altered in any way during the editing process. The quality of the edit has been guaranteed, with the assumption that our suggested changes have been accepted and the text has not been further altered without the knowledge of our editors.

MANUSCRIPT TITLE

Efficacy of traditional herbal medicine treatment based on pattern identification for idiopathic Parkinson's disease: A protocol for systematic review and meta-analysis

AUTHORS

Purumea Jun, HuiYan Zhao, Ojin Kwon, and Jung-Hee Jang

ISSUED ON

October 28, 2021

JOB CODE

BJAWV_15_3

THE EDITAGE Promise QUALITY GUARANTEE

Vikas Narang

Vikas Narang
Chief Operating Officer - Editage

editage

Editage, a brand of Cactus Communications, offers professional English language editing and publication support services to authors engaged in over 1300 areas of research. Through its community of experienced editors, which includes doctors, engineers, published scientists, and researchers with peer review experience, Editage has successfully helped authors get published in internationally reputed journals. Authors who work with Editage are guaranteed excellent language quality and timely delivery.

GLOBAL :
+1(833) 979-0061 | request@editage.com

KOREA :
02-3478-4396 | submit-korea@editage.com

CACTUS

IMPACT SCIENCE impact.science

researcher.life

CACTUS LIFE SCIENCES lifesciences.cactusglobal.com
